# Effects on and transfer across the blood-brain barrier in vitro—Comparison of organic and inorganic mercury species

**DOI:** 10.1186/s40360-016-0106-5

**Published:** 2016-12-15

**Authors:** Hanna Lohren, Julia Bornhorst, Romy Fitkau, Gabriele Pohl, Hans-Joachim Galla, Tanja Schwerdtle

**Affiliations:** 1 Department of Food Chemistry, Institute of Nutritional Science, Univeristy of Potsdam, Potsdam, Germany; 2 Institute of Biochemistry, University of Muenster, Muenster, Germany

**Keywords:** Organic mercury, Inorganic mercury, Methylmercury, Thiomersal, Mercuric mercury, In vitro blood-brain barrier model

## Abstract

**Background:**

Transport of methylmercury (MeHg) across the blood-brain barrier towards the brain side is well discussed in literature, while ethylmercury (EtHg) and inorganic mercury are not adequately characterized regarding their entry into the brain. Studies investigating a possible efflux out of the brain are not described to our knowledge.

**Methods:**

This study compares, for the first time, effects of organic methylmercury chloride (MeHgCl), EtHg-containing thiomersal and inorganic Hg chloride (HgCl_2_) on as well as their transfer across a primary porcine in vitro model of the blood-brain barrier.

**Results:**

With respect to the barrier integrity, the barrier model exhibited a much higher sensitivity towards HgCl_2_ following basolateral incubation (brain-facing side) as compared to apical application (blood-facing side). These HgCl_2_ induced effects on the barrier integrity after brain side incubation are comparable to that of the organic species, although MeHgCl and thiomersal exerted much higher cytotoxic effects in the barrier building cells. Hg transfer rates following exposure to organic species in both directions argue for diffusion as transfer mechanism. Inorganic Hg application surprisingly resulted in a Hg transfer out of the brain-facing compartment.

**Conclusions:**

In case of MeHgCl and thiomersal incubation, mercury crossed the barrier in both directions, with a slight accumulation in the basolateral, brain-facing compartment, after simultaneous incubation in both compartments. For HgCl_2_, our data provide first evidence that the blood-brain barrier transfers mercury out of the brain.

## Background

Mercury (Hg) is present in the environment due to both natural sources and anthropogenic activity. It exists as elemental Hg as well as inorganic and organic compounds, all incorporating different toxicological properties [1, 2]. Human exposure to organic species mainly results from the consumption of contaminated fish or seafood in the form of methylmercury (MeHg), the most frequently occurring organic species in the aquatic food chain [3]. The use of the ethylmercury (EtHg) containing preservative thiomersal in medical preparations, including vaccines, represents a non-dietary route of human exposure towards organic Hg species [4]. In contaminated terrestrial food, Hg is mainly present as inorganic Hg [2]. In 2012, in accordance to the Joint FAO/WHO Expert Committee on Food Additives (JECFA) [2] the European Food Safety Authority (EFSA) Panel on Contaminants in the Food Chain established a tolerable weekly intake (TWI) of 4 μg/kg body weight (b.w.) for inorganic Hg [5]. Based on new epidemiological data, the EFSA Panel reevaluated for MeHg the provisional tolerable weekly intake (PTWI) of 1.6 μg/kg b.w. (JECFA [6]) and established a TWI of 1.3 μg/kg b.w.. High fish consumers may exceed this TWI by up to six fold [5].

The central nervous system (CNS) represents the major target organ of organic Hg species exposure [7]. Thiomersal shows a higher or at least similar toxicity compared to MeHg in brain associated cells in vitro (e.g. [8, 9]). However, the toxic potential of MeHg under in vivo conditions seems to be higher, which might be a consequence of different disposition kinetics [10].

The transport of MeHg across the blood-brain barrier to the brain side is well described in literature, while EtHg and inorganic Hg are not adequately characterized regarding their entry into the brain. Based on in vitro as well as in vivo studies an active transport mechanism of MeHg as a cysteine complex (MeHg-S-Cys) across the blood-brain barrier via the L-type neutral amino acid transport (LAT) system has been proposed [11–14]. It has to be noted that the transport of MeHg across the blood-brain barrier involves both, uptake into and efflux from brain endothelial cells. Heggland et al. postulated that LAT does not seem to be involved in the efflux of MeHg in vitro but that MeHg is transferred out of brain endothelial cells as a complex with glutathione (GSH), thereby using GSH-transporters [15]. In 2013 Zimmermann et al. postulated a LAT system based uptake of MeHg-S-Cys as well as EtHg-S-Cys into C6 glioma cells, whereas MeHg and EtHg enter the cells by other mechanisms [16]. Besides this active, energy dependent transfer of organic Hg species, transfer mechanisms can be passive depending on the Hg species [17]. Following intramuscular injection of 20 μg thiomersal in mice, EtHg as well as inorganic Hg were identified in brain tissue indicating that EtHg can pass the blood-brain barrier [18]. In vivo, a rapid metabolism of EtHg to inorganic Hg is described [10, 19]. Thus, because of a possible dealkylation of the organic compounds as well as an oxidation of elemental Hg, e.g. resulting from the outgassing of amalgam fillings, inorganic Hg has to be taken into account, when investigating mechanisms of Hg species dependent transfer into/out of the brain and Hg species induced neurotoxicity [20, 21]. Toimela et al. demonstrated transfer differences between mercury chloride (HgCl_2_) and methylmercury chloride (MeHgCl) within an in vitro blood-brain barrier model composed of rat brain endothelial cells accompanied with glia cells and neuronal SH-SY5Y cells as target cells [22]. The authors concluded from cytotoxic effects on the neuronal cells in the brain-facing compartment that MeHgCl passed the barrier model because it exerted cytotoxic effects on the neuronal cells, while barrier-building cells were not affected. HgCl_2_ did not cause any toxicity to neuronal cells, as long the barrier cells were not affected [22].

In the present study, a well-characterized primary porcine in vitro blood-brain barrier model was applied to compare the effects of MeHgCl, thiomersal and HgCl_2_ on the barrier as well as to characterize their transfer properties across this barrier model. In contrast to existing studies, we focused on both transfer into as well as transfer out of the brain.

## Methods

### Primary blood-brain barrier cell culture model

Isolation, cultivation and cryopreservation of primary porcine brain capillary endothelial cells (PBCECs) from brain tissue of freshly slaughtered pigs (from the slaughterhouse) was performed as previously described [23, 24]. On day in vitro (DIV) 2, PBCECs were thawed and seeded on rat tail collagen-coated Transwell^®^ filter inserts with microporous polycarbonate membranes (1.12 cm^2^ growth area, 0.4 μM pore size; Corning, Wiesbaden, Germany) in plating medium (Medium 199 Earle supplemented with 10% newborn calf serum, 0.7 μM L-glutamine, 100 μg/mL gentamycin, 100 U/mL penicillin, 100 μg/mL streptomycin (all Biochrom, Berlin, Germany)) according to literature [25]. After 2 days of proliferation (DIV 2–4) PBCECs reached confluence and differentiation was induced by replacing the plating medium with serum-free culture medium (Dulbecco’s modified Eagle’s medium/Ham’s F12 (1:1) with 4.1 mM L-glutamine, 100 μg/mL gentamycin, 100 U/mL penicillin, 100 μg/mL streptomycin (Biochrom) and 550 nM hydrocortisone (Sigma Aldrich, Deisenhofen, Germany)) [26]. According to manufacturer’s information, this medium contains 15.75 mg/L (100 μM) L-cysteine*HCl and 17.24 mg/L (115 μM) L-methionine. All experiments were started after another 2 days of differentiation (DIV 6). The PBCECs built up a tight monolayer on the rat tail collagen-coated polycarbonate membranes comparable to the epithelium of a brain microvessel. The collagen coating on the filter corresponds to the basal membrane in vivo. Epithelial cells seeded on this surface in vitro develop tight junctions ensuring the polarity between apical and basolateral cell membranes. Thus, in this fully developed in vitro model of the blood-brain barrier the apical (upper) compartment refers to the lumen of the vessel in vivo and therefore mimics the blood side. Vice versa, the basolateral compartment represents the parenchymal side of the blood-brain barrier and mimics the brain side in vivo [23].

### Preparation of Hg species stock solutions

Stock solutions of MeHgCl (>99.9% purity, Sigma Aldrich, Deisenhofen, Germany), thiomersal (>97% purity, Sigma Aldrich) and HgCl_2_ (>99.999% purity, Sigma Aldrich) were prepared in sterile distilled water shortly before each experiment. Thiomersal is well known to release ethylmercury (EtHg) in aqueous solutions [27].

### Cytotoxicity testing

For the evaluation of cytotoxic effects of the Hg species on PBCECs the neutral red uptake assay was performed to quantify the lysosomal integrity. This endpoint has been shown before to be both applicable to assess viability of compounds in PBCECs in general [25] and to be suitable to assess cytotoxicity of mercury species e.g. in human astrocytes [8]. The cellular uptake of neutral red depends on the cell’s capacity to maintain pH gradients, which strongly depends on the cellular ATP level [28]. Fonfria et al. demonstrated both, decreased intracellular ATP levels as well as decreased mitochondrial activity, in murine cerebellar granule cells following incubation of high MeHgCl and HgCl_2_ concentrations [29], providing additional evidence that lysosomal integrity is a suitable marker to assess Hg species induced cytotoxicity. Briefly, PBCECs were cultivated in rat tail collagen-coated 96 well culture plates under the same conditions as the cells seeded on Transwell^®^ filter inserts. Cells were exposed to the respective Hg species on DIV 6. After 72 h the neutral red uptake assay was carried out according to literature [25].

### Cellular bioavailability

PBCECs were cultivated in rat tail collagen-coated 24 well culture plates and cultured according to the cultivation in Transwell^®^ filters. After 72 h incubation of the respective Hg species, PBCECs were washed twice with PBS (100 mM NaCl, 4.5 mM KCl, 7 mM Na_2_HPO_4_, 3 mM KH_2_PO_4_ (all Sigma Aldrich); pH 7.4) and incubated with 120 μL lysis buffer (RIPA-buffer; 0.01 M Tris, pH 7.6, 0.15 M NaCl, 0.001 M EDTA, 1% sodium desoxycholate, 0.1% (all Sigma Aldrich)) for 15 min on ice. After scrapping off and sonication, the suspension of lysed cells was centrifuged at 10 000 x g for 20 min at 4 °C. Total cellular Hg content was quantified by inductively coupled mass spectrometry (ICP-MS; Agilent 8800 ICP-QQQ, Agilent Technologies Deutschland GmbH, Boeblingen, Germany) in an aliquot of the supernatant. The Bradford assay was used to determine the cellular protein level.

### Barrier integrity, capacitance and transfer measurements

The organic (0.01–3 μM) and the inorganic (0.01–100 μM) Hg species were applied on DIV 6 either to the apical (blood-facing) or to the basolateral (brain-facing) compartment of the in vitro blood-brain barrier model or to both compartments simultaneously by replacing 10% of the culture medium with fresh Hg species containing medium in the respective compartment. Barrier integrity was monitored during 72 h of incubation by measurement of the transendothelial electrical resistance (TEER) using the cellZscope (nanoAnalytics, Münster, Germany). Furthermore, the capacitance, which is related to the plasma membrane surface area, was recorded online (cellZscope; nanoAnalytics, Münster, Germany). Wells with TEER values of ≥ 600 Ω x cm^2^ and a capacitance of 0.45–0.6 μF/cm^2^ were used for the experiments providing a confluent PBCEC monolayer with tight barrier properties. Standard deviation of TEER values was < 18% and for capacitance values < 11% (not shown).

For transfer studies aliquots of both compartments were taken after 0, 3, 6, 24, 48 and 72 h in case of incubation on one side. In case of application on both sides simultaneously the first sample was collected after 1.5 h. The total Hg amount in these samples was quantified by ICP-MS. Hg transfer was calculated as % in relation to the total Hg content in both compartments.

### ICP-MS analysis

For quantification of the total Hg content by ICP-MS, aliquots of the transfer studies and cellular bioavailability studies were diluted in a solution of 5% HNO_3_ + 5% HCl (v/v, both suprapur, Merck KGaA). An external calibration (1–150 ng/L; Hg standard for ICP, TraceCERT^®^, Fluka, Deisenhofen, Germany) was prepared in the same solution. Samples and calibration standards were incubated with rhodium (final concentration 10 ng/L; Merck KGaA, Darmstadt, Germany) as internal standard. A MicroMist nebulizer was used for sample introduction and gas flows were applied as follows: 15 L/min cool gas, 0.9 L/min auxiliary gas and 1 L/min nebulizer gas. The method exhibits a limit of detection of 0.8 ng/L and a limit of quantification of 2.9 ng/L calculated by the calibration method of the German Standard DIN standard 32645 [30].

## Results

### Cytotoxicity and bioavailability in primary PBCECs

**Fig. 1:**
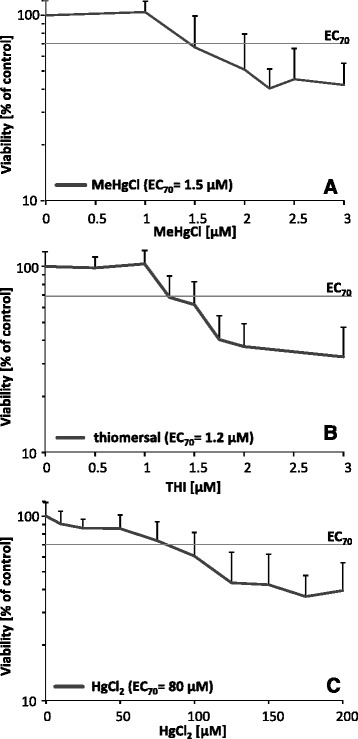
Cytotoxicity of MeHgCl (**a**), thiomersal (**b**) and HgCl_2_ (**c**) in PBCECs after 72 h incubation. Cytotoxicity was determined by a decrease of the lysosomal integrity measured by neutral red uptake. PBCECs were cultivated in rat tail collagen-coated 96 well culture plates under the same conditions as the cells seeded on Transwell^®^ filter inserts. Shown are mean values of at least 3 independent determinations with 6 replicates + SD

Cell viability was assessed in confluent PBCECs by lysosomal integrity. The organic Hg species MeHgCl (EC_70_ 1.5 μM) and thiomersal (EC_70_ 1.2 μM) exerted higher cytotoxic effects as compared to inorganic HgCl_2_ (EC_70_ 80 μM), with thiomersal being the most cytotoxic compound (Fig. 1a – c). In accordance to the higher cytotoxicity of the respective organic species, PBCECs showed higher intracellular total Hg concentrations following exposure to subcytotoxic but transfer relevant concentrations (0.01, 0.1 and 1 μM) of the organic mercury species (Table 1).

**Table 1:** Cellular bioavailability of MeHgCl, thiomersal and HgCl_2_ in PCPECs after 72 h incubation

	Concentration [μM]	Cellular Hg [μg Hg/mg protein]
Control	0	n.d.
MeHgCl	0.01	0.010 ± 0.004
	0.1	0.135 ± 0.051
	1	0.807 ± 0.059
thiomersal	0.01	0.021 ± 0.002
	0.1	0.084 ± 0.023
	1	0.511 ± 0.107
HgCl_2_	0.01	0.004 ± 0.000
	0.1	0.010 ± 0.002
	1	0.047 ± 0.033

PBCECs were cultivated in rat tail collagen-coated 24 well culture plates under the same conditions as the cells seeded on Transwell® filter inserts. Data represent mean values of at least 2 independent determinations with 2 replicates each ± SD

### Evaluation of the barrier integrity and the capacitance

The applied well-characterized cell culture model of the blood-brain barrier is built up by fully differentiated PBCECs cultivated on Transwell^®^ filter inserts between two fluid compartments filled with cell culture medium [23, 24]. The upper apical compartment refers to the blood side in vivo, whereas the lower basolateral compartment represents the brain side. The impact of the mercury species on the barrier integrity was assessed by online monitoring of the transendothelial electrical resistance (TEER) during the entire transfer experiment, since the electrical resistance correlates with the tightness of the barrier.

**Fig. 2:**
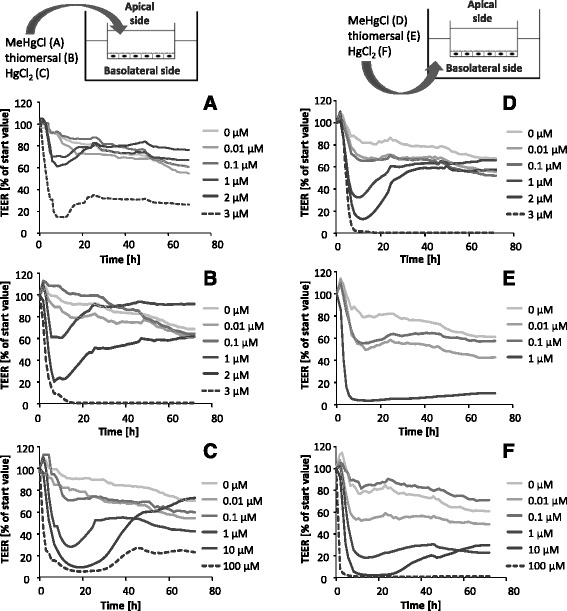
Effect of MeHgCl, thiomersal and HgCl_2_ on the barrier integrity of the PBCEC monolayer after application in the basolateral compartment (blood side, **a** – **c**) and in the apical compartment (brain side, **d** – **f**) for 72 h. Barrier integrity was determined by online measurement of the TEER. Shown are mean values, expressed as % of start value, of at least 3 independent determinations with 2 replicates minimum with SD < ± 20% (not shown)

Comparing the apical and the basolateral application a higher sensitivity of the barrier to all species following brain side incubation was observable (Fig. 2a – f). The barrier integrity was massively disturbed below TEER values of 30% of the start value, leaving a weakened barrier with 10–300 Ω xcm^2^ (depending on the start value). Following incubation on the apical side (blood-facing side), 3 μM MeHgCl or thiomersal, and 100 μM HgCl_2_ disrupted the tightness of the barrier (Fig. 2a – c). In case of 2 μM thiomersal as well as 1 and 10 μM HgCl_2_ incubation, early barrier disturbing effects were also visible. Nevertheless, the TEER values increased again at later time points, indicating a recovery of the barrier functions.

After basolateral incubation (brain-facing side) the barrier integrity is irreversibly affected at 3 μM MeHgCl, 1 μM thiomersal and 1 μM HgCl_2_ (Fig. 2d – f). 1 and 2 μM MeHgCl strongly decreased TEER values to 15–35% but allowed a reconstitution of the barrier integrity to about 70%.

**Fig. 3:**
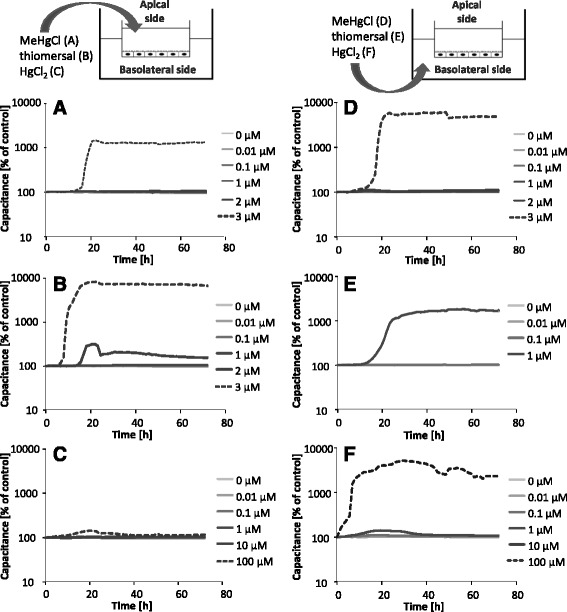
Effect of MeHgCl, thiomersal and HgCl_2_ on the electrical capacitance after application in the apical compartment (blood side, **a** – **c**) and in the basolateral compartment (brain side, **d** – **f**) for 72 h. Shown are mean values of at least 3 independent determinations with 2 replicates minimum with SD < ± 10% (not shown)

Since the electrical capacitance is related to the plasma membrane surface area, online monitoring of the electrical capacitance within the in vitro model of the blood-brain barrier indicates cytotoxic effects (Fig. 3a – f). The apical and the basolateral incubation of 3 μM MeHgCl lead to a strong increase of the electrical capacitance, pointing towards an enlarged cell volume, apoptotic or detached cells. Following apical incubation of thiomersal the electrical capacitance started to increase at a concentration of 2 μM, whereas the application of 1 μM on the basolateral side of the endothelial cells increased the capacitance dramatically. A concentration of 100 μM HgCl_2_ caused no effect on the capacitance after apical application, but an increase of capacitance following basolateral incubation.

### Transfer across the in vitro model of the blood-brain barrier

Transfer across the blood-brain barrier was assessed at 0.01, 0.1 and 1 μM of the Hg species. The barrier tightness was not affected by these concentrations, except for a basolateral incubation with 1 μM thiomersal or HgCl_2_.

**Fig. 4:**
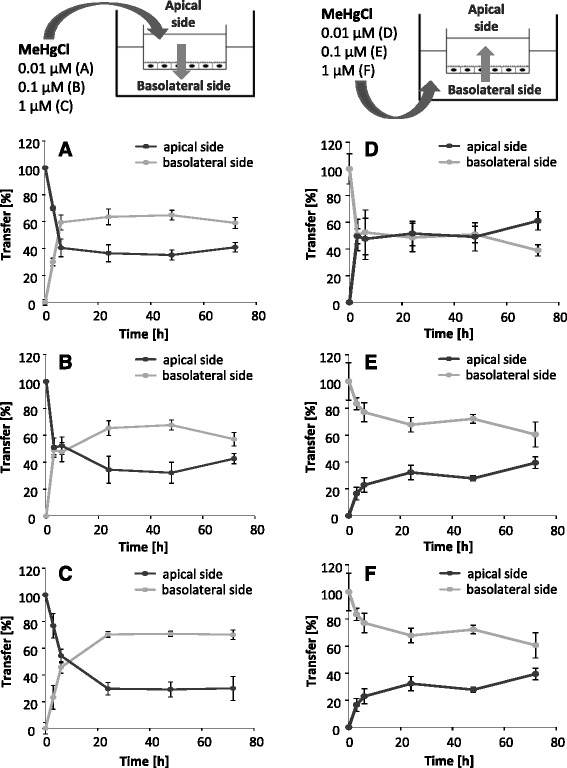
Transfer of MeHgCl after incubation in the apical compartment (blood side, **a** – **c**) and in the basolateral compartment (brain side, **d** – **f**) for 72 h. Data are expressed as % of the whole Hg amount quantified in both compartments. Shown are mean values of at least 3 independent determinations with 3 replicates ± SD

The application of MeHgCl in the apical compartment led to similar Hg transfer rates for all concentrations (Fig. 4a – c). Within the first 6 h, the Hg content increased to 50–60% of the total applied mercury in the basolateral compartment and reached a maximum of 75% in the following 66 h. The Hg amount on the apical side decreased correspondingly. After incubation in the basolateral compartment, the application of 0.01 μM MeHgCl led to a rapid transfer towards the blood side, reaching 60% of the applied Hg amount in this compartment. In case of 0.1 and 1 μM MeHgCl application, Hg was slowly transferred out of the basolateral compartment. Nevertheless, the total Hg content on the apical side of the endothelial cells did not exceed the Hg amount in the brain side (Fig. 4d – f).

**Fig. 5:**
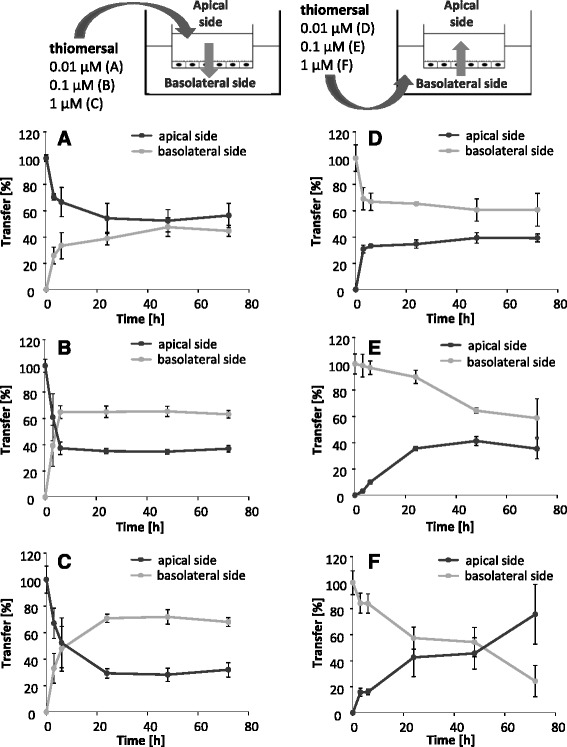
Transfer of thiomersal after incubation in the apical compartment (blood side, **a** – **c**) and in the basolateral compartment (brain side, **d** – **f**) for 72 h. Data are expressed as % of the whole Hg amount quantified in both compartments. Shown are mean values of at least 3 independent determinations with 3 replicates ± SD

The apical incubation of 0.01 μM thiomersal resulted in an equalization of the Hg content in both compartments (Fig. 5a). The Hg transfer behavior from the apical to the basolateral side following incubation with 0.1 and 1 μM thiomersal was comparable to the Hg transfer following MeHgCl exposure (Fig. 5b, c). In case of basolateral incubation with 0.01 and 0.1 μM thiomersal, the Hg contents in both compartments converge. The disturbance of the barrier integrity at 1 μM led to equally distributed Hg contents in both compartments (Fig. 5d – f).

**Fig. 6:**
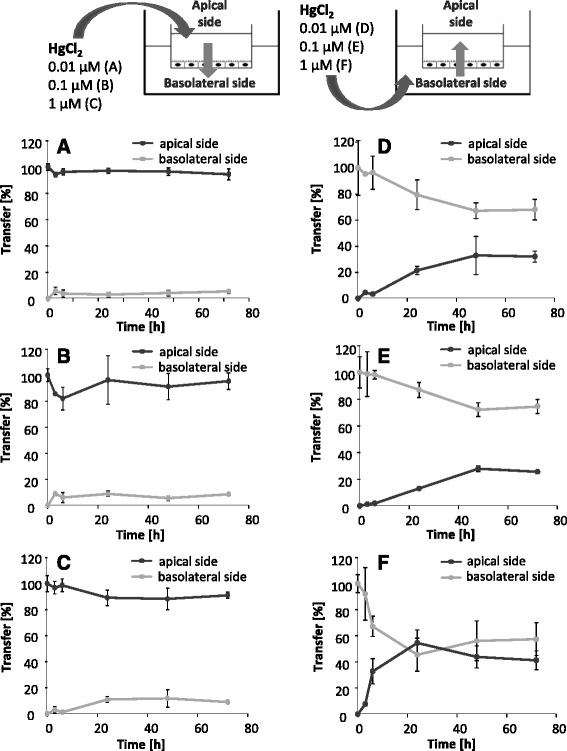
Transfer of HgCl_2_ after incubation in the apical compartment (blood side, **a** – **c**) and in the basolateral compartment (brain side, **d** – **f**) for 72 h. Data are expressed as % of the whole Hg amount quantified in both compartments. Shown are mean values of at least 3 independent determinations with 3 replicates ± SD

The transfer following inorganic HgCl_2_ exposure clearly differs from the organic mercury species. Thus, after 72 h of exposure in the apical compartment nearly the whole Hg amount was still present in this compartment (Fig. 6a – c). In contrast, application on the basolateral side led to Hg transfer rates of up to 30% towards the acceptor compartment (Fig. 6d, e). The concentration exchange following 1 μM HgCl_2_ incubation reflected the affected tightness and leakage of the barrier (Fig. 6f).

**Fig. 7:**
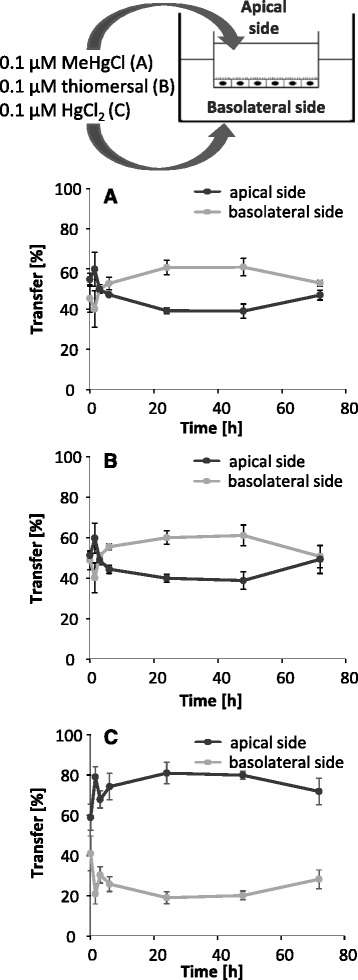
Transfer of 0.1 μM MeHgCl (**a**), 0.1 μM thiomersal (**b**) and 0.1 μM HgCl_2_ (**c**) after incubation in the apical compartment (blood side) and in the basolateral compartment (brain side) simultaneously for 72 h. Data are expressed as % of the whole Hg amount measured in both compartments

In a further approach, each 0.1 μM MeHgCl, thiomersal and HgCl_2_ were applied on both sides simultaneously (Fig. 7a – c). The slight Hg accumulation in the basolateral compartment within the first 48 h and the Hg concentration balancing after 72 h of exposure to MeHgCl and thiomersal do not indicate an active transfer mechanism from the apical to the basolateral side of the endothelial cells. In case of simultaneous HgCl_2_ application in both compartments, Hg accumulated in the apical compartment.

## Discussion

The primary target organ of organic Hg species mediated toxicity is the CNS [31]. In literature, the understanding of transfer mechanisms into the brain is limited to MeHg; first evidence exists for a comparable carrier mediated transfer mechanism for EtHg [11, 16]. To our knowledge, a possible efflux of organic and inorganic mercury species out of the brain side has yet not been evaluated. Although inorganic Hg does not seem to be able to cross the blood-brain barrier [32], it is present in the brain due to dealkylation of organic species or an oxidation of elemental Hg [18, 21]. Thus, the elucidation of an efflux of inorganic Hg besides the organic compounds is of special interest.

According to previous cytotoxicity studies in a human astrocytoma cell line [8], organic MeHgCl and thiomersal exerted in the present study stronger cytotoxic effects in the barrier building PBCECs as compared to inorganic HgCl_2_. The bioavailability studies, performed in subcytotoxic but transfer relevant concentrations, give evidence that this enhanced cytotoxicity is a result of a higher cellular Hg content following exposure to the organic compounds.

The TEER measurements clearly demonstrate differences in barrier integrity following basolateral incubation (brain side) as compared to apical (blood side) incubation. Barrier leakage caused by 3 μM of the organic and 100 μM of the inorganic species applied in the apical compartment correlated with the respective cytotoxic effects. In contrast, after basolateral application, the barrier was more sensitive towards all mercury species, but especially to inorganic HgCl_2_. This enhanced sensitivity of the barrier integrity towards basolateral HgCl_2_ application is not in line with the results of the cytotoxicity and bioavailability data, showing more than 10 fold higher cellular Hg concentrations after exposure to the organic species than to HgCl_2_.

Since in literature most studies investigating Hg mediated neurotoxicity focus on MeHg or EtHg, the effects of inorganic Hg species on brain endothelial cells are poorly understood. Oppedisano et al. demonstrated a more effective inhibition of the glutamine/amino acid transporter (ASCT2) [33], a carrier located at the abluminal site of the blood-brain barrier with brain to endothelium orientation, by HgCl_2_ as compared to MeHgCl [34]. Fonfria et al. reported an alteration in neuronal glutamate transport associated with the excitatory amino acid transporter (EAAT3), a transporter with the same orientation as ASCT2 [33], induced by HgCl_2_ [29]. It might be possible, at least because of steric advantage of the Hg^2+^ ion as compared to organic Hg species, that inorganic Hg ions generally exert stronger effects on transporters located to the abluminal side of the blood-brain barrier. Thus, a disruption of the barrier tightness following basolateral incubation might be a consequence of disturbed transport properties and homeostasis. This hypothesis needs to be proven in further studies.

Our results indicate a Hg transfer following organic MeHgCl and thiomersal exposure across the in vitro model of the blood-brain barrier towards the basolateral side of the endothelial cells. This is in contrast to inorganic HgCl_2_, which does not seem to be transferred towards the basolateral compartment in the case of an intact barrier. These results correspond to different in vivo studies, postulating a transfer of organic species into the brain [10, 18]. Since the respective culture media in the present study contains cysteine, a complex formation of organic species with cysteine is conceivable. These complexes have been shown before to enter the brain via a neutral amino acid carrier by mimicking the structure of methionine. Nevertheless, it has also been taken into account that the medium contains substantial levels of methionine, which has been discussed to disturb the transport of the respective organic mercury cysteine complexes [11, 16]. We can also not fully exclude that the applied lipophilic organic Hg species are not fully complexed to thiols and thus are capable to transfer across cell barriers by diffusion, whereas the hydrophilic inorganic HgCl_2_ is not expected to diffuse through cell membranes [11, 12]. Since total Hg blood concentrations of 0.6–30 μg/L (0.03–0.15 μM) are related to dietary Hg exposure in a population group with a wide range of seafood consumption and Hg is mainly present as MeHg in marine food [35], the application of 0.01 and 0.1 μM represent concentrations of physiological relevance. Burbacher et al. reported total blood Hg concentrations of 8–18 ng/mL (0.04–0.09 μM) 2 days after oral exposure of 20 μg/kg MeHg as MeHg hydroxide in infant monkeys. The intramuscular injection of one single thiomersal dose in humans (20 μg/kg) led to total blood Hg concentrations of 6–14 ng/L (0.03–0.07 μM) [10]. The concentrations of HgCl_2_ used are of experimental importance to suggest that Hg^2+^ apparently tends to transfer more easily from the basolateral to the apical side of the blood-brain barrier model than from the apical to the basolateral side.

In a second approach, we investigated for the first time Hg transfer from the basolateral side to the apical side of the PBCECs (efflux). After application of the organic compounds in the basolateral compartment, a Hg transfer out of the brain-facing compartment was evident. Nevertheless, efflux rates were lower as compared to influx rates. Surprisingly, Hg transfer data after basolateral HgCl_2_ incubation indicate for an Hg efflux. These results are in accordance to the simultaneous application on both sides. Whereas the simultaneous application of organic species led to a slight accumulation on the basolateral side within the first 48 h, the inorganic species clearly accumulated in the apical compartment. Since a dealkylation of organic species in the brain, especially the in vivo observed rapid conversion of EtHg to inorganic Hg [18], as well as the oxidation of elemental Hg result in the presence of inorganic Hg in the brain, these findings might indicate a possible detoxification mechanism in Hg mediated neurotoxicity. Based on the efflux of inorganic Hg, the blood-brain barrier might protect the target organ brain from Hg induced neurotoxic damage. The results of the simultaneous incubation of the organic compounds argue for diffusion as transfer mechanism. According to uptake studies in brain endothelial cells [15] an active carrier mediated transport to the brain side as well as to the blood side can be excluded for MeHgCl and thiomersal.

## Conclusions

Applying a well-characterized primary blood-brain barrier model, mercury influx and efflux were assessed, after exposure towards organic MeHgCl, thiomersal, and inorganic HgCl_2_. Our data indicate for the organic mercury species a transfer in both directions, with a slight accumulation on the basolateral side of the endothelial cells (brain side), thereby arguing for diffusion as transfer mechanism. HgCl_2_ was not able to cross the in vitro barrier towards the basolateral side, but partially transferred out of the basolateral compartment following basolateral incubation. Thus, the blood-brain barrier might play a role in preventing the target organ brain from Hg induced neurotoxic effects.

## Abbreviations

CNS: Central nervous system
DIV: Day in vitro
EC: Effective concentration
EFSA: European Food Safety Authority
EtHg: Ethylmercury
GSH: Glutathione
HgCl_2_: Hg chloride
JECFA: Joint FAO/WHO Expert Committee on Food Additives
MeHg: Methylmercury
MeHgCl: Methylmercury chloride
MeHg-S-Cys: MeHg as a cysteine complex
PBCECs: Primary porcine brain capillary endothelial cells
PTWI: Provisional tolerable weekly intake
TEER: Transendothelial electrical resistance
TWI: Tolerable weekly intake
